# Coproducing a new scale with young people aged 10–24 years: a protocol for the development and validation of the Youth Loneliness Scale (YLS)

**DOI:** 10.1136/bmjopen-2024-097497

**Published:** 2025-07-22

**Authors:** Delia Fuhrmann, Laura Riddleston, Lily Verity, Iqra Alam, Lizet Chavez, Jasmine Conway, Amilah Niaz, Ayla Pollmann, Pamela Qualter, Poppy Spowage, Lauren Turner, Wahida Walibhai, Jennifer Y F Lau

**Affiliations:** 1 King’s College London, London, UK; 2 Queen Mary University of London, London, UK; 3 The University of Manchester, Manchester, UK

**Keywords:** Psychometrics, Adolescents, MENTAL HEALTH, PUBLIC HEALTH, QUALITATIVE RESEARCH

## Abstract

**Abstract:**

**Introduction:**

The high prevalence of loneliness in young people, aged 10–24 years, is increasingly recognised as an urgent global health concern. The experience of loneliness is linked to a wide range of adverse physical and mental health outcomes. A lack of loneliness scales that can accurately capture the authentic experiences of young people has hampered progress in our understanding of the aetiology and sequelae of youth loneliness, as well as the development of preventative policies and interventions. Here, we provide a protocol for developing and validating an age-sensitive loneliness scale for young people aged 10–24 years: the Youth Loneliness Scale (YLS). The scale is designed to measure loneliness in the general population of young people in the UK.

**Methods and analysis:**

The scale is coproduced with young people from design to dissemination. The scale development process follows a three-phased, multistep approach that includes item development, scale construction and scale evaluation. Item development is achieved via deductive (literature review) and inductive methods (arts workshops and focus groups), as well as a Delphi survey of experts (by profession and experience) for initial refinement. The scale is then constructed via pretesting items in cognitive interviews with young people, and exploratory testing for preliminary evaluation and refinement. Finally, the scale is administered in confirmatory testing, where a full psychometric evaluation is provided.

**Ethics and dissemination:**

The project was approved by the Queen Mary University of London Research Ethics Committee (Reference: 2024-0231-341) as the lead site and subsequently endorsed by the University of Manchester Research Ethics Committee. The YLS scale and results of its psychometric evaluation will be published open-access. The protocol provided here will allow researchers to evaluate the final scale generated against the plans set out. We also encourage the use and adaptation of the protocol to develop age-sensitive loneliness scales for other populations.

**STRENGTHS AND LIMITATIONS OF THIS STUDY**

- The Youth Loneliness Scale (YLS) is coproduced with young people from design to dissemination, ensuring that it reflects the authentic experiences of young people.
- The YLS development process follows a multistep approach, generating qualitative and quantitative data to inform item generation, scale construction and scale evaluation.
- YLS items are generated via a mix of focus groups and arts workshops. This approach allows for both verbal and non-verbal expressions of loneliness. Arts workshops also provide participants with opportunities for connection.
- The YLS covers both relationship-specific items (friends, family/caregivers) and general items. This balances the need for concrete items that can inform research and practice, with the need for general items that may be particularly useful for short scales.
- The YLS is designed to measure loneliness in the general population of young people in the UK. For use in other populations, the YLS may need adaptation first.

## Introduction

 Loneliness is a significant public health issue. It is defined as the negative feelings that arise when we are dissatisfied with the quality or quantity of our social relationships.^1^ Loneliness predicts long-term health problems,^2^ poorer quality of life^3^ and mortality.^4^

One of the age groups most likely to be affected by loneliness is young people: 45% of 10–15-year-olds and 59% of 16–24-year-olds in the UK report feeling lonely ‘often’ or ‘some of the time’.^5^ These peaks in loneliness in youth are thought to reflect age-normative changes.^6^ Navigating significant transitions—including beginning or finishing secondary school, entering employment or starting university—can bring considerable upheaval and uncertainty to social relationships. These external pressures occur alongside internal developmental shifts in social cognition,^7^ including a heightened sensitivity to peer acceptance and rejection,^8 9^ making feelings of social disconnection more salient at this life stage. Getting trapped in what may initially be age-typical feelings of loneliness can become a more prolonged chronic experience maintained by cognitive-behavioural factors. This, in turn, may contribute to social avoidance and become a risk factor for developing mental health conditions, including anxiety and depression.^10 11^

Young people have been calling for interventions that address youth loneliness and protect their well-being and mental health.^12^ However, formal and developmentally tailored interventions that help young people manage their loneliness and subsequent mental health difficulties are emerging but still scarce.^13^ Young people also struggle to find resources to manage loneliness through statutory services or third-sector organisations.^12^ This gap in support arises, in part, because progress in researching mechanisms of and interventions for youth loneliness is slow.

Progress in youth loneliness research has been hindered by a paucity of developmentally sensitive measures of loneliness that can capture loneliness experiences across youth and identify individuals needing support.^14^ Measurement of loneliness across age groups has been an ongoing challenge^15^; these problems are thought to be particularly acute in youth measures.^14 16^ Current measures of youth loneliness are suboptimal in several ways. First, measures used in large-scale population studies of youth loneliness sometimes include only a single item (‘How often do you feel lonely?’).^17^ There are concerns that such direct measures yield imprecise loneliness estimates due to the stigma young people feel about loneliness,^18 19^ and/or demand characteristics. Second, even where measures include multiple items, often they have been developed with little input from young people.^14^ The absence of lived experience to guide item generation may mean that the constructed scales fail to capture the authenticity and distinctiveness of lonely feelings experienced in youth—which in turn may (a) conceptually mask developmental differences that may exist in the nature and experiences of loneliness between young people and working age/older adults and (b) methodologically, undermine the validity and reliability of loneliness reports. Third, measures currently used in youth either lack psychometric evaluation for youth or have been evaluated only for specific age groups within youth.^14^ Items of a commonly-used loneliness measure, the University of California, Los Angeles (UCLA) loneliness scale,^20^ are age-variant,^16^ suggesting differences in the way that different age groups interpret items. That, again, raises questions about the validity of loneliness measures, particularly in younger-aged samples. Fourth, loneliness is usually probed only through ratings of frequency (eg, ‘How often do you feel lonely?’) with little reference to timeframes, limiting contextual precision,^21^ and limited consideration of other important dimensions,^10^ such as duration, intensity and emotional impact. Taken together, the current evidence indicates that the call from young people for interventions that address youth loneliness cannot be met without first creating measures of youth loneliness that are fit for purpose.

The Youth Loneliness Scale (YLS) project’s overarching objective is to develop, evaluate and disseminate a developmentally sensitive measure of loneliness for young people aged 10–24 years living in the UK. We select this broad age range to allow us to track age-linked continuities and changes in how loneliness is experienced across the full set of developmental transitions in youth. The aim of this protocol is to make our design and analysis plan transparent, allowing for a better evaluation of the final scale. The preprint of this protocol has been updated as the research progresses, but changes can be checked against previous versions of the protocol available on the OSF: https://osf.io/preprints/psyarxiv/2g5ba. The first version of the protocol was uploaded before the start of data collection. A second aim is to provide a template protocol that can be adapted and validated by researchers working with young people growing up in other countries and cultural contexts. We hope this will help us measure and understand the diversity and universal aspects of loneliness experiences in youth. For the design of the project, we followed recommendations for best practices in measurement development^22^,^24^ but also leveraged the expertise of key stakeholders, including young people themselves. The YLS has been coproduced with young people from design to dissemination, ensuring the YLS reflects young people’s experiences, needs and priorities.^25^ This also reduces reliance on the existing literature, which might be limited, and supports content and face validity.^26^ A final aim of our work is to generate a protocol for a new qualitative approach that helps young people communicate their experiences. We use a combination of traditional focus group topic guide prompts with participatory arts-based techniques to generate scale items to help young people express the authentic emotional, cognitive and behavioural features of loneliness in verbal and non-verbal ways.^27^

## Methods and analysis

Based on best practices in the field and recommendations for rigorous measure development,^22^,^24^ we follow a three-phase process comprising item development, scale construction and scale evaluation (see table 1). Each phase contains several steps and sub-steps. Before the start of phase 1 (item generation), conceptual work was completed, including reviewing existing scales, reviewing qualitative work (published and, where available, grey literature) with youth that included a discussion of what loneliness is, and identifying the design needs for the scale (see above). Research materials associated with the protocol (eg, topic guides for the qualitative work and code for sample size calculations) can be retrieved from https://osf.io/4qvnh/. Steps 1–3 were completed at the time of submission of this protocol. Steps 4 and 5 were not completed and should be read as a preregistration. The first version of this protocol (dated 7 August 2023) was uploaded to the OSF before the start of data collection.

**Table 1:** Overview of the development process for the Youth Loneliness Scale

Phase	Step	
Phase 1: Item development	Step 1: Item generation	
	Deductive item generation (literature review)	I=14
	Inductive item generation (workshops/focus groups)	I=66 N_Workshops_=51 N_FocusGroups_=93
	Step 2: Item refinement	
	Expert feedback using the Delphi method	I=80 N_YoungPeople_=48 N_Professionals_=24 T=3
Phase 2: Scale construction	Step 3: Pre-testing	
	Feedback via cognitive interviews with young people	I=30 N=30
	Step 4: Exploratory testing	
	Preliminary evaluation and refinement of the scale using an exploratory sample and test–retest reliability assessment	I=25 Target N=250 T*=*2
Phase 3: Scale evaluation	Step 5: Confirmatory testing	
	Full psychometric evaluation of the scale using a confirmatory sample	I~20 (exact number TBC after Step 4) N~700 (exact number TBC after Step 4

Notes: Steps 1–3 were completed at the time of submission. They informed the design of the protocol presented here. Steps 4 and 5 were not completed at the time of submission and should be read as a preregistration.

I, number of items; N, number of participants; T, number of time points; TBC, to be confirmed.

The samples for steps 1–3 were diverse in terms of ethnicity and somewhat skewed towards higher parental education and income levels but with reasonable absolute participant numbers across education and income brackets. There were more females than males overall. Detailed participant characteristics are reported in the Supplementary Information: https://osf.io/4qvnh/.

### Patient and public involvement

The YLS is coproduced with young people from design to dissemination, ensuring the YLS reflects young people’s experiences, needs and priorities. Young people are reimbursed for their time contributing to the project. Young people are involved at the following levels of the project: IA and JC were coinvestigators on the grant proposal, and IA, JC and WW are coauthors of this paper. They have contributed to all project stages by providing feedback and leading the coproduction work. Around six young people are advisors in our young person advisory group, who give feedback on research design, materials and dissemination. Two of these young people are seconded to our steering committee on a rotating basis to provide guidance and oversight. Additional groups of young people support ongoing work on the YLS as summer students, contributing, for example, lived experience advice or supporting recruitment and the production of dissemination outputs.

### Phase 1: item development

#### Step 1: item generation

In line with best practices,^22 23^ we aimed to generate around five times the number of items we wish to retain in the final scale. As we anticipated generating a 10-item to 20-item scale, we aimed to generate 50–100 items in step 1. We generated 80 items in total: 66 from our qualitative data and 14 from existing measurement scales. We followed recommendations for item characteristics,^22^ including generating simple, unambiguous items suitable for Likert responses.^28^

We combined inductive and deductive methods to generate these items. For inductive item generation, we conducted workshops with young people combining traditional qualitative focus group approaches with participatory arts approaches. Previous work suggests that focus groups and participatory arts approaches reveal complementary information.^27^ In these, we explored (A) behaviours, emotions and cognitions associated with loneliness and (B) relevant dimensions of these experiences (frequency, intensity, duration, emotional impact). For example, participants were asked to create an improvisation scene of a situation where someone might feel lonely, followed by a discussion about why they had improvised that situation. We held workshops in Manchester and London (where the research team members are based). Between the two sites, we held two workshops each for age groups 8–11 years and 12–15 years, and three workshops each for age groups 16–18 years and 19–24 years. In total, 51 young people participated in the workshops. Age groups were determined based on educational transitions in the UK. Using narrower age groups was more efficient when presenting prompts and generating conversations between participants. Although the final scale is intended for use in 10–24 years, we deliberately included some younger participants in this initial qualitative work. Including 8 and 9 year olds in the item-generation process helped us identify language and experiences that were comprehensible and relevant to young people across a broad age range. This strengthened the scale’s capacity to resonate with the lower end of our target age range (10–12 years), which can be particularly challenging.^14 29^ To capture diverse experiences of loneliness, we recruited via a wide range of organisations in both London and Manchester, including universities, summer schools, local youth organisations, charities and community action projects working with young people. The workshops followed a topic guide codeveloped with young people (see https://osf.io/4qvnh/ for research materials). The workshops were facilitated by a professional artist who led creative activities and was assisted by a trained researcher. Both in-person and online focus groups supplemented arts workshops to reach populations who could not attend the in-person workshops. In-person focus groups were conducted in London and Manchester via schools, colleges and youth organisations with young people aged 8–18 years. Online focus groups were conducted with young people aged 19–24 years. In total, 93 young people took part in the focus groups.

All sessions were audio recorded with consent and transcribed. Transcripts of the workshops and focus groups were analysed using conventional content analysis.^27^ The workshop transcripts were coded by LR and LV, who assigned codes to participants’ responses. Codes developed through that initial analysis were then applied to the focus group transcripts, and additional codes were generated where participants reported new insights. Codes were developed using participants’ phrasing; over 300 items were initially generated. The research team continually refined these items through group discussions at moderation meetings.^30^ Criteria used to assess items during these meetings were guided by DeVellis and Thorpe^22^ and included combining similarly phrased items into a single item that captured the overarching meaning, ensuring items were straightforward to understand and appropriate for the target demographic, and applicable across all age groups. We also omitted items that were double-barrelled, required reverse-scoring, used double negatives, were likely to be ‘leading’ or were likely to elicit little variation in responses. This resulted in a list of 66 loneliness statements for further refinement in step 2.

For deductive item generation, we conducted a content analysis of the relevant theoretical and empirical literature, mainly qualitative studies and existing scales, to generate a broad pool of potential items. We examined existing measures to identify items not covered in our inductive item generation. A total of 14 items were extracted to be included alongside inductively generated items in step 2.

Apart from item generation, step 1 data were also used to inform the scale’s coverage. For example, the literature review indicated the importance of the cognitive, behavioural and emotional facets of loneliness.^31^ Loneliness was also thought to vary in intensity, duration and frequency; in line with previous accounts of loneliness.^21 32^

#### Step 2: item refinement

We sought feedback from two groups of experts on the items developed in step 1. The first group comprised 48 experts by experience (young people who have experienced loneliness), and the second group comprised 24 professional experts (academics working on loneliness and/or youth development and/or psychometrics; and other stakeholders such as charities or practitioners working with young people). Using the Delphi method,^33^ we collected experts’ anonymous opinions across three rounds of feedback. Each round lasted up to 4 weeks, with the survey open for at least 2 weeks. Instructions for the Delphi survey design were developed and refined with input from our summer students and young person advisory group (see https://osf.io/4qvnh/ for the research materials). For the Delphi survey, we provided experts with an operational definition of loneliness (“(…) the negative experience that happens when a person feels unhappy with their relationships with other people”).

In round 1, experts were asked to rate the face validity of the items (“How relevant do you think these items are to young people’s experience of loneliness?”) on a 5-point Likert scale ranging from ‘very irrelevant’ to ‘very relevant’. Items were presented in blocks of 5 (with two blocks per page). After two blocks, a free text question was displayed asking for comments on the items’ clarity (for experts by experience), technical quality (for professional experts) and any other feedback. There was also a final free text question that allowed experts to suggest new items that we had not already included. Finally, we also sought input regarding the time frame to consider when asking questionnaire users to reflect and report on their loneliness experiences. 17 professionals and 45 experience experts participated in round 1.

In rounds 1 and 2 of our Delphi study, we set our level of agreement at 70%, that is, ≥70% of participants needed to rate an item as either ‘slightly relevant’ or ‘very relevant’ for it to be deemed that agreement had been reached that the item had face validity. The same applies in the opposite direction; ≥70% of participants needed to rate an item as either ‘slightly irrelevant’ or ‘very irrelevant’ to be deemed that agreement had been reached that the item did not have face validity. In rounds 1 and 2 of the study, data from each expert group were analysed separately.

In round 1, professional experts agreed that 57 items had high face validity, and experience experts agreed that 41 items had high face validity. Qualitative feedback indicated that some of these items would benefit from further refinement (I=15 for professional experts, I=8 for experience experts). These items were edited based on the feedback and then included in round 2 of the study for re-rating.

Professional experts failed to reach agreement on the face validity of 23 items, and experience experts failed to reach agreement on the face validity of 39 items. These items were therefore included in round 2 of the study for re-rating. Again, where qualitative feedback indicated that an item was unclear, the item was edited before inclusion in round 2.

In both groups, six items (a mixture of items that had and had not reached agreement on validity) were excluded following round 1. These items were thought to lack face validity, or qualitative feedback indicated irreconcilable issues with the items (ie, not applicable for neurodivergent youth). Additional items proposed by experts were reviewed for inclusion in round 2, with four new items subsequently included.

In total, 41 items for the professional experts, and 31 items for the experience experts, were carried straight forward to round 3 of the study, as agreement on face validity had been achieved and no modifications to the items based on feedback had been made.

In round 2, we asked the expert groups to re-rate the face validity of items that failed to reach agreement in round 1 (I=33 items presented to professional experts and I=43 presented to experience experts), plus new items generated from qualitative feedback in round 1 (I=4). We provided feedback to experts on the findings from round 1 (eg, ‘Based on your feedback, this item was changed from ‘I find changes to my friend group difficult to cope with’ to ‘I find it difficult to cope with disruptions to my friendship groups’. Last time, 41% of your group agreed that this item was very relevant to young people’s experiences of loneliness.’). Again, free-text questions asked for feedback on the items after every ten items. Finally, we asked experts’ opinions on the dimensionality of the questionnaire ratings, for example, whether we should ask for reports of loneliness duration, intensity and/or frequency. 15 professional and 45 lived-experience experts participated in round 2.

During round 2, professional experts agreed that 20 items had high face validity, and experience experts agreed that 35 items had high face validity. As in round 1, some of these items were edited based on qualitative feedback before being carried forward to round 3. The professional experts agreed that 17 items either lacked face validity, agreement on face validity was not reached or the item was decided to be unclear. The experience experts agreed that 12 items either lacked face validity, agreement on face validity was not reached or the item was decided to be unclear. These items were therefore excluded.

Following round 2, we combined results from both expert groups to determine which items to take forward into round 3. Across rounds 1 and 2, 56 items that both expert groups rated as having high face validity were retained for inclusion in round 3, and the remaining 16 items were excluded. Both groups recommended asking about the time frame of the ‘past month’. In terms of the most important dimension to ask about, both groups recommended asking about intensity as the most important, followed by frequency.

In round 3, all items that reached agreement across groups for high face validity across rounds 1 and 2 were sent out to experts (I=56), who were asked to rank the items in order of their importance for measuring loneliness in young people. Based on usability feedback from young people, we provided experts with four boxes (‘most important’, ‘kind of important’, ‘kind of unimportant’, ‘least important’). Participants were asked to drag and drop items into these boxes. Afterwards, they were asked to sort items in order of importance within each box. 17 professional and 41 experience experts participated in round 3.

Based on qualitative feedback from rounds 1 and 2, we also asked experts’ opinions on the most important types of relationships to cover in the scale (eg, general, friendships and family members/caregivers). In quantitative and qualitative feedback, experience experts reported friends and family as the most important relationships to ask about. They also noted that these items are concrete and easy to understand. Professional experts preferred the questionnaire to include general items that do not reference specific relationships. To balance these demands, we included both concrete items referring to friends and family/caregivers and general items. We also asked experts’ opinions on how many scale points to use (4 to 7-point scale). Both groups preferred a 5-point Likert scale.

Rankings were calculated by creating ranked lists of items for all participants and individually for the two expert groups. We used the following statistics to determine the final set of items: M_O_—median ranking overall, M_Y_—median ranking young people, M_P_—median ranking professionals, IQR_O_—IQR overall. We considered items with M_O_<28 (top half of rankings) and additionally one item with M_o_=30 (‘I don’t feel supported by my family/caregivers’) to ensure equal coverage of friendship and family domains. This yielded 33 items. Three items were excluded because they were similar in content to other, higher-ranking, items (table 2).

**Table 2:** Items retained at the end of phase 1

Items retained at the end of phase 1	M_O_	M_Y_	M_P_	IQR_O_
I feel alone*	5	4.5	7.5	13
I don’t have anyone to talk to when something is bothering me*	14	16	9	17
I don’t feel loved by my family/caregivers†	6	4	27.5	23
I feel like there are not many people in my life who care about me†	16.5	16.5	16.5	18
I feel left out of things*	17	14	27	24
I don’t feel like I have supportive people in my life*	19	18	20	20
I feel like an outsider*	18	16	21	31
I feel like people don’t want to hang out with me†	27	26	31	16
I don’t feel accepted by my family/caregivers†	17	13.5	26.5	29
There isn’t anyone I can be myself with*	24	24	18	27
I feel like my family/caregivers don’t understand how I feel†	22	22	20.5	17
I don’t feel wanted†	16	14	22	20
I feel like I have no one to count on	21	21	22	19
I don’t have a friend I feel close to*	24	26	8.5	22
I don’t feel like I have anyone I can depend on*	21	23	13.5	24
I don’t have anyone that I trust*	20	19.5	25	25
I feel ignored by my friends†	13	12.5	37	25
I feel my family/caregivers will judge me if I talk about my feelings†	23	20.5	47	28
I don’t feel very close to my family/caregivers*	24	24	28.5	26
I don’t feel important to my family/caregivers*	17	15	38	25
I feel like my friends are fed up with me†	19	17	44	32
I feel like I'm missing out on important friendships or relationships with other people†	26	27	24	27
I can’t find a friend when I need one	27	29	17	17
I don’t feel close to the people around me	27	30	15	22
I feel separate from my friends†	26	28	13	2
I feel like my friends don’t understand how I feel†	23	21	33	28
I feel isolated	22	23	15	30
I don’t feel emotionally supported by my family/caregivers†	30	31	28	24
I don’t feel like I belong with my family/caregivers	19	19	26	25
I feel like my friends are leaving me out†	22	27	15	32

* Retained for step 4.

† Retained for step 4 but edited in line with feedback from step 3—the final wording is shown here.

IQR_O_, IQR overall; M_O_, median ranking overall; M_P_, median ranking professionals; M_Y_, median ranking young people.

### Phase 2: scale construction

#### Step 3: pretesting

To ensure that items are phrased in ways that are meaningful to young people, we conducted cognitive interviews^34^ with young people (N=30), starting with an initial set of 30 items. To ensure items are suitable for the general population, we recruited young people with a range of loneliness experiences. In these interviews, we administered the draft items to young people and asked them to verbalise their thoughts when completing the ratings (see https://osf.io/4qvnh/ for research materials). We took an iterative approach and clarified or dropped items four times across step 3. Five items were dropped due to insufficient clarity, not being appropriate for all ages or not being interpreted as intended.

The interview schedule was also updated to focus on the readability of items that previous participants had difficulty with. We conducted 20 interviews (individual, group, online and in-person) with 30 young people. Interviews were conducted with young people aged 10–11 (N=7), 12–15 (N=6), 16–18 (N=9) and 19–24 years (N=8).

At the end of step 3, 11 items were retained that captured general feelings of loneliness (eg, ‘I feel left out of things’), seven items that were family/caregiver-specific (eg, ‘I don’t feel very close to my family/caregivers’) and seven items that were friendship-specific (eg, ‘I feel ignored by my friends’).

#### Step 4: exploratory testing

Step 4 onwards was not completed at the time of submission. The following information, therefore, serves as a preregistration of our research plans. Using an online survey platform, we will test the refined items from step 3 (I=25, target N=250). The survey is administered twice, 2 weeks apart,^35 36^ to assess test–retest reliability. Note that we originally planned to assess test–retest reliability in step 5 but moved it to step 4 to obtain this information earlier in the scale development process. Participants complete all 25 items, with each item being rated according to three dimensions:

- Intensity: “thinking about the past month, how bothered have you felt by this?” (not at all bothered, slightly bothered, somewhat bothered, very bothered, extremely bothered).
- Frequency: “thinking about the past month, how often did you feel like this?” (never, hardly ever, sometimes, often, always).
- Duration: “how long did the feeling last?” (I didn’t feel like this, less than a day, days, weeks, a month or longer).

We will conduct analyses both for a composite score averaging across these dimensions and each dimension individually.

For each time point separately, we will examine Cronbach’s alpha, inter-item correlations, item-discrimination index, corrected item-total correlation, as well as the distribution and variance of responses for each item. Problematic items with low consistency with other items will be removed. Exploratory factor analysis (EFA) will be employed to test the dimensionality of the scale and further identify poorly performing items and/or dimensions for removal. For this, we will use parallel analysis, as implemented in the *psych* package^37^ in *R*: fa.parallel(data, fa=‘fa‘, n.iter=100). We will repeat this analysis with polychoric correlations as a robustness check.^38^ The results of the fa. parallel will also be compared with the comparison data (CD) and sequential model tests (SMT) methods implemented in *EFAtools*^39 40^ as a robustness check. The EFA will then be fit with rotate=‘oblimin’, implementing an oblique rotation, to allow for correlations between factors.^41^ Cross-loading items or items with poor loadings^42^ will be removed unless there is a strong theoretical rationale for keeping them.

The following criteria will be applied to determine items for consideration for removal:

- Items an inter-item correlation below 0.2.^43^
- Items where the Cronbach’s alpha is above 0.9 if the item is deleted (which may indicate redundancy).^44^
- Items with an item-discrimination index below 0.2.^43^
- Items with an item-total correlation below 0.3^45^
- Items with factor loadings below 0.4.^41^
- Items with cross-loadings above 0.3.^41^
- Items with a difference between the loading on the primary and alternative factors below 0.2.^41^

Where the resulting scale yields a solution with fewer than four items per factor, we will consider removing that factor. This is because a factor with fewer than four items can pose issues with identification and reliability.^46^

We will generate scale scores for preliminary reliability analyses using the reduced items. Reliability is ‘the reproducibility or consistency of scores from one assessment to another’. Provisional internal consistency will be assessed using McDonald’s coefficient omega and Average Variance Extracted using *semTools* in R.^47^ Provisional split-half and odd-even reliability will be assessed using the splitHalf function in *psych*.^37^ Test–retest reliability will be assessed using the intraclass correlation coefficient (ICC) using *irr*^48^ with the following specifications: icc(ratings, model=c(‘twoway’), type=c(‘agreement’), unit=c(‘single’)).^49^

A minimum sample size of 250 was determined based on Monte Carlo simulations for continuous items using maximum likelihood estimation and 1500 replications. We used a three-factor solution with 11, 7 and 7 indicators (representing ‘general’, family/caregiver’ and ‘friendship’ domains, respectively), with factor loadings λ=0.50, ε=0.75. This showed that a sample size of N=250 provided a good model fit, even when accounting for 10% missing data. This also maintains the recommended ratio of respondents to items of at least 10:1.^23 42^ A sample size calculation using the *ICC.Sample.Size* package^50^ also indicates that the sample size exceeds what is needed to determine test–retest reliability with 90% power, alpha=0.05.

### Phase 3: scale evaluation

#### Step 5: confirmatory testing

For confirmatory testing of the final set of items, we will recruit a UK sample of youth 10–11 years, 12–15 years, 16–18 years and 19–24 years, from a range of ethnic backgrounds and representative in terms of gender. Quotas for this will be taken from the UK census (https://census.gov.uk/). Items will be administered using an online survey platform. Participants will also complete other questionnaire measures, scores on which will be compared with the YLS to assess convergent and divergent validity (table 3). Scales were selected for their psychometric properties and validation in relevant age ranges (see table 3 for details). Few scales were validated for the entire age range (10–24 years). Therefore, a young person-coinvestigator (IA) reviewed them for face validity.

**Table 3:** Additional questionnaires included for validation in the survey at step 5

Construct	Scale	Reliability and validity
Loneliness	Single item loneliness measure^17^	Convergent validity with UCLA Loneliness scale scores in samples aged 10–15 years and 16+ years in the UK (G=0.877, p<0.00).^60^
	Three-Item Loneliness Scale for children^61^* Three-Item Loneliness Scale^62^*	Good internal consistency (α=0.85) in UK sample aged 10–15 years.^60^ Acceptable internal consistency (α=0.76) in UK sample aged 16+.^60^
	UCLA-20^63^	Excellent internal consistency (α=0.92). US-based sample consisted of university students with age not reported.^63^
Depression	Patient Health Questionnaire modified for Adolescents (PHQ-A)^64^	Diagnostic validity in 13–18 year-olds with a clinical interview (56% of adolescents who were identified as having a mental disorder by PHQ-A were diagnosed with a mental disorder a mental health professional). Mean age of patients in the US-based validation sample was 15.90±1.24 years.^64^
General anxiety	Generalised Anxiety Disorder^65^	Convergent validity with the Paediatric Anxiety Rating Scale (*R*=0.65, p*<*0.05). Mean age of patients in US-based validation sample was 14.8±2.8 years.^66^
Social anxiety	Screen for Child Anxiety Related Emotional Disorders (SCARED)–Social Anxiety Disorder subscale^67^† Screen for Adult Anxiety Related Disorders (SCAARED)–Social Anxiety Disorder subscale^68^†	SCARED: Convergent validity with child trait subscales (r=0.73, p<0.001) and child state subscale (r=0.73, p<0.001) of the State-Trait Anxiety Inventory for Children. Mean age of patients in the US-based validation sample was 14.4±2.3 years.^67^ SCAARED: Social Anxiety subscale demonstrated good to excellent internal consistency (α=0.86–0.97). Mean age of participants in US-based validation sample was 20.2±1.9 years.^68^
Well-being	Short Warwick-Edinburgh Mental Well-being Scale^69^	Demonstrated acceptable internal consistency in a sample of 10–16-year-olds based in Denmark (α=0.78)^70^ and good internal consistency in a sample of Norwegian adolescents, aged 15–21 years (α=0.88).^71^
Introversion/ extroversion	Items from the international personality item pool^72^	Demonstrated good internal consistency (α=0.87) among a Scottish student sample aged 17–61 years.^72^

* In line with validation studies, different versions will be administered to participants aged 10–15 and 16+.

† In line with validation studies, different versions will be administered to participants aged 10–17 and 18+.

Confirmatory factor analysis (CFA) will be used to determine whether the factor structure determined at step 4 holds in an independent sample. CFA models will be fit using *lavaan*^51^ in R. Models will be fitted using maximum likelihood estimation with robust (Huber-White) SEs and a scaled test statistic. Missing data will be estimated using the full information maximum likelihood method. Model fit will be assessed using the *χ*^2^ test, the RMSEA (Root Mean Square Error of Approximation) and its confidence interval, the Comparative Fit Index (CFI) and the standardised root mean square residual (SRMR). Good fit will be defined as RMSEA <0.05, CFI >0.97 and SRMR <0.05 and acceptable fit as RMSEA=0.05–0.08, CFI=0.95–0.97 and SRMR=0.05–0.10.^52^ Configural, metric and scalar measurement invariance will be assessed to determine the factor structure in different age groups (10–11 years, 12–15 years, 16–18 years, 19–24 years) and genders. Nested models will be compared using the delta χ^2^, RMSEA and CFI using the criteria set out in.^53^

Tests of reliability from step 4 (except for test–retest reliability) will be repeated for the confirmatory sample and for each age group. Additional tests of validity will be completed. Validity is ‘the degree to which evidence and theory support the interpretations of test scores entailed by the proposed uses of tests’. Initial assessments of validity will have taken place in steps 1–4. Step 5 will allow for an assessment of the final scale. Convergent and divergent validity will be assessed by comparing scores on the new measure to those on relevant scales listed in table 3. A strong correlation between scales (r>0.5) will be taken to indicate convergent validity of scales measuring the same construct.^54^ A correlation below 0.9 between measures of different constructs will be taken to suggest acceptable discriminant validity.^55^ We expect positive strong correlations between the YLS and other measures of loneliness. We expect positive, but weaker correlations between the YLS and measures of depression, general anxiety, social anxiety and introversion. We expect negative and weaker correlations between the YLS and well-being. Analyses of convergent and divergent validity will be conducted using suitable R packages, such as the *measureQ* package.^55^

The final sample size for confirmatory testing will be determined based on the final number of items retained at step 4. Sample sizes will be adjusted to test multigroup models (across four age groups). Preliminary sample size calculations using Monte Carlo approaches and *semPower*^56^ indicate that 700 participants exceed the sample size needed to run a 4-group multigroup CFA model, comparing, for example, configural and metric measurement invariance models (alpha=0.05, power=0.90).

## Discussion

Youth loneliness is a significant public health issue in many countries worldwide,^57^ yet existing measures do not necessarily capture the lived experiences of young people. Here, we provide a protocol for developing a developmentally sensitive scale measuring youth loneliness between the ages of 10 and 24 years in the general population in the UK. With this protocol, the scale is developed, refined and evaluated in a multistep approach following best practices in scale development.^22^,^24^

The protocol has two key novel features:

First, it has been and will be coproduced with young people from design to dissemination, ensuring that it captures their authentic experiences of loneliness. Previous scales used to measure youth loneliness were developed with little input from young people.^14^ The absence of lived experiences to guide the development of scales is not unique to loneliness or youth populations. Still, it may mean that the current scales fail to capture the authentic and distinctive experiences of loneliness in youth.^58 59^

Second, it combines established qualitative approaches (eg, qualitative focus groups) with arts-based approaches, ensuring that we can tap into non-verbal expressions of loneliness. Generating a comprehensive pool of items in the initial steps is essential to obtain a well-functioning scale at the end of the development process.^22^ To achieve this, we use a combination of traditional qualitative focus groups and participatory arts-based approaches to help young people better describe the authentic emotional, cognitive and behavioural features of loneliness. Different qualitative methods can appeal to different groups of young people. Different methods are also useful in accessing different information within the same young person.^27^ We find that arts-based workshops have the additional benefit of bringing young people together in person and building a rapport in the group through creative activities. Interspersing creative activities with focus-group-style questions also helps maintain participants’ stamina for discussing loneliness. At the same time, online focus groups were conducted with those aged 18 years and above because this age group was more challenging to recruit for the in-person workshops and showed a preference for spending time focused on discussion rather than creative activities.

Recruiting and retaining young people as research volunteers for the project is a priority. We are working with young people to develop a recruitment strategy that engages young people with diverse experiences and backgrounds. We are also working with our partners’ networks (eg, a school and UK charities) to identify schools, colleges, universities, training colleges, companies and businesses in geographically, culturally and socioeconomically diverse areas. Finally, we reimburse young people for their time and efforts through gift vouchers.

The protocol provided here evidences our development process, enabling a better evaluation of our final scale. It can also be adapted for other populations and contexts, enabling better measurement of loneliness or other constructs worldwide. Where target populations are similar to ours, it may be sufficient to translate and validate our final scale in that population. Where the target population is likely to have different experiences and expressions of loneliness (eg, in contexts without the widespread use of social media), we recommend using a multistep approach like the one outlined here, generating new items and then iteratively refining them to ensure validity and reliability of the final scale.

## Ethics and dissemination

The project was conducted in accordance with the Declaration of Helsinki, approved by the Queen Mary University of London Research Ethics Committee (Reference: 2024-0231-341) as the lead site and subsequently endorsed by the University of Manchester Research Ethics Committee. Across phases of data collection, young people aged 18 years and above gave informed consent via a paper or online consent form. For those under 18 years, legal guardians were required to provide consent while assent was obtained from the young person.

The YLS scale and its psychometric evaluation will be published open-access. Pathways to disseminate the scale will be codesigned with young people, with input from our young person’s advisory group, steering committee members and other relevant stakeholders (academic researcher colleagues, non-academic partners, practitioners, policy-makers and funders).
