# Supplementary material for: Coproducing a new scale with young people aged 10–24 years: a protocol for the development and validation of the Youth Loneliness Scale (YLS)
Source: BMJ Open. 2025 Jul 22;15(7):e097497. doi: 10.1136/bmjopen-2024-097497 (PMC12306216; doi:10.1136/bmjopen-2024-097497)
Supplement: Supplementary file 1 [file bmjopen-2024-097497.reviewer_comments.pdf]

PEER REVIEW HISTORY

BMJ Open publishes all reviews undertaken for accepted manuscripts. Reviewers are asked to complete a checklist review form and are provided with free text boxes to elaborate on their assessment. These free text comments are reproduced below.

ARTICLE DETAILS

Title (Provisional)

Co-producing a new scale with young people aged 10 – 24 years: A protocol for the development and validation of the Youth Loneliness Scale (YLS)

Authors

Fuhrmann, Delia; Riddleston, Laura; Verity, Lily; Alam, Iqra; Chavez, Lizet; Conway, Jasmine; Niaz, Amilah; Pollmann, Ayla; Qualter, Pamela; Spowage, Poppy; Turner, Lauren; Walibhai, Wahida; Lau, Jennifer Y F

VERSION 1 - REVIEW

**Reviewer**	**1**
**Name**	**Sivaraman, Sruthi**
**Affiliation**	**Kristu Jayanti College**
**Date**	**07-Feb-2025**
**COI**	**None**

Look into the abstract

**Reviewer**	**2**
**Name**	**Novak, Lukas**
**Affiliation**	**Palacky University Olomouc, Social Health Institute**
**Date**	**25-Feb-2025**
**COI**	**None**

Dear Editor,

thank you very kindly for the opportunity to review this protocol. I think that the development of a new loneliness measure focused on assessing loneliness in young people can be a meaningful contribution to the field. Overall, the quality of the protocol is very high. However, there are several points I invite authors to think about or incorporate into their protocol to potentially improve the quality of the resulting study.

First, the authors state that: „Youth Loneliness Scale (YLS) project's overarching objective is to develop, evaluate, and disseminate a developmentally sensitive measure of loneliness for young people aged 10-24 years living in the UK. We select this broad age range to allow us to track age-linked continuities and changes in how loneliness is experienced across the full set of developmental transitions in youth." Although I understand the author's intention to capture the degree of loneliness across a wide age group of young people, I think that it would be desirable to provide more arguments for the lower and upper bound of the selected age group of interest. In more detail, why 10 and not e.g. 8 years old children, are creating a lower bound of the age group of interest, and why do 24 and not 26 form the upper bound of the age group of interest?

Second, in Step 4, the authors say: „The survey will be administered twice, two weeks apart, to assess test-retest reliability." What rationale do authors have for selecting a two-week period for test-retest and not, e.g., one week?

Third, the authors say that: „Problematic items with low reliability, variability or consistency with other items will be removed." I understand that it might be challenging to set up thresholds for some metrics, e.g., variability of scores and that to decrease researchers' degrees of freedom, it might be advisable to define thresholds for, e.g., internal consistency, factor loadings, etc. This might increase transparency for item selection in step 4. I would also ask authors to specify more clearly, what the authors mean by poorly performing dimensions.

Fourth, the authors state that: „Exploratory Factor Analysis (EFA) will be employed to test the dimensionality of the scale and further identify poorly performing items and/or dimensions for removal. For this, we will use parallel analysis, as implemented in the psych package [32] in R." Although I am very happy that the authors specified what method they will use to extract a number of factors, I think that some additional arguments for using parallel analysis must be provided – especially given the fact that there exist many methods for factor extraction (see the following simulation study for more details: https://doi.org/10.1037/met0000200). Relatedly, I would recommend authors use at least two methods for factor extraction because it adds credibility to a number of extracted factors if results from both methods will yield the same results. On the OSF page, I could not find code for the simulation study determining minimal sample size. Please consider sharing your simulation code.

Fifth, the authors say: „Test-retest reliability will be assessed using the Intraclass Correlation Coefficient (ICC) using irr. " I appreciate very much that the authors are specific regarding how ICC will be calculated. I would however, strongly recommend to authors what type of ICC will be used since this can be definitely specified in advance. See this canonical article for more details: https://doi.org/10.1016/j.jcm.2016.02.012

Sixth, authors states: „invariance will be assessed to determine the factor structure in different age groups and genders." I would recommend including the exact age categories

that will be tested in MI. Relatedly, what reasons do authors have for omitting scalar invariance testing?

Finally, the authors expect that the scale will have three dimensions. This expectation needs to be based on some theoretical framework underlying this expectation. Thus, I suggest that the authors state what theoretical framework guided them to think about their scale as having three dimensions.

Relatedly, please explain (at least to me in your response) why you think that a common cause theory will generate your data and why you do not think about relationships between items as they are mutually interacting with each other in a network. See e.g., this article: https://doi.org/10.1080/1047840X.2020.1853461.

**Reviewer**	**3**
**Name**	**McKenna-Plumley, Phoebe**
**Affiliation**	**Queen's University Belfast**
**Date**	**21-May-2025**
**COI**	**None**

Thank you for the opportunity to review this manuscript. This is a timely and valuable scale development effort which will surely be of interest to a range of researchers and practitioners. The scale development process appears appropriate and has many strengths (the use of co-production with young people throughout and the focus on intensity, frequency, and duration of loneliness, for example) and the statistical plan seems generally thorough. I have some points for clarification which are outlined below; I hope that these can be helpful for the authors.

COMMENTS:

This topic is timely and the rationale behind creating this scale is strong. A well-constructed scale for young people would be a valuable addition to research and practice around loneliness and it is very encouraging to see meaningful engagement with young people in creating this scale.

Page 4: “Three young people are coinvestigators on the project and have co-authored the protocol; young people’s feedback is also integrated into the protocol via a Young Person Advisory Group. Finally, additional groups of young people contribute to the project during the summer, offering their perspectives at different stages of the project”: It is surely valuable to have these insights from young people throughout project development and conduct. However, this section doesn’t make it very clear how the roles of these three groups (co-investigators, advisory group, and additional groups) differ – this information is

expanded in the Discussion (page 13-14), so perhaps some of this could be added/moved to this initial description of the different roles.

Could the authors comment on their rationale for specifically focusing on 10–24-year-olds, rather than a narrower or wider age range? Relatedly, it seems that some workshop participants were younger (8-11 years) - is there a reason why some younger participants were also included in the development work but won't be considered a relevant age group for the final scale?

Page 6: "The research team continually refined these items through group discussions at moderation meetings": It could be helpful to understand the types of decisions that were made through these discussions – i.e. what were the criteria leading to items being discarded at this stage?

Perhaps the authors could briefly comment on characteristics of the young people who contributed to scale development beyond age and gender? This does not need to be exhaustive and confidentially is vital, but a narrative description of other demographic characteristics that were included/missing in these groups would be useful. For example, on page 8, it is noted that one item was removed as it was not applicable for neurodivergent youth – with this in mind, it would be useful to understand how diverse the overall group were in ways beyond age and gender, given that their insights were very instrumental to the development process.

Page 9: "Both groups recommended asking about the time frame of the 'past month'. In terms of the most important dimension to ask about, both groups recommended asking about intensity as the most important, followed by frequency.": For readers unfamiliar with other loneliness measures, it might be helpful to comment briefly on how much this aligns or differs from other common loneliness scales and whether the authors anticipate any implications for making comparisons with other youth scales or adult loneliness measures.

Could the authors comment on their decision to have items focusing on specific relationship types (family/caregiver and friends) within the items, rather than having all non-specific items (like "I don't feel wanted")? Some of these items may be hard to respond to as they depend on the availability of these relationships (e.g. "I feel ignored by friends" - a 'Strongly disagree' response could mean that a person is satisfied with their friends or that they don't have friends to feel ignored by). I appreciate that these relationships were considered most important by the expert groups, but perhaps it is worth commenting on the strengths and weaknesses of including specific relationships within the scale.

The planned EFA and CFA processes appear to be generally thorough and appropriate. Some additional information would be valuable on certain points - perhaps the authors could specify the values that would constitute high cross-loading or poor loading items, and the factor extraction method and rotation for EFA.

In Table 3, could the authors include a specification of the direction and potentially the magnitude of the correlations they expect to observe between these variables and the YLS to decide that convergent and divergent validity have been achieved?

The Discussion is thorough and clearly identifies the strengths and need for the authors' approach, as well as guiding future work in terms of scale adaptation.

I hope these comments can be useful for the authors.

VERSION 1 - AUTHOR RESPONSE

Reviewer: 1
Dr. Sruthi Sivaraman, Kristu Jayanti College
Comments to the Author: Look into the abstract

We have revised the abstract as follows:

Abstract (page 2):

Introduction: *The high prevalence of loneliness in young people, ages 10 – 24 years, is increasingly recognised as an urgent global health concern. The experience of loneliness is linked to a wide range of adverse physical and mental health outcomes. A lack of loneliness scales that can accurately capture the authentic experiences of young people has hampered progress in our understanding of the aetiology and sequelae of youth loneliness, as well as the development of preventative policies and interventions. Here, we provide a protocol for developing and validating an agesensitive loneliness scale for young people aged 10-24 years: the Youth Loneliness Scale (YLS). The scale is designed to measure loneliness in the general population of young people in the United Kingdom.*

Methods and Analysis: *The scale is co-produced with young people from design to dissemination. The scale development process follows a three-phased, multi-step approach that includes item development, scale construction and scale evaluation. Item development is achieved via deductive (literature review) and inductive methods (arts workshops and focus groups), as well as a Delphi survey of experts (by profession and experience) for initial refinement. The scale is then constructed via pre-testing items in cognitive interviews with young people, and exploratory testing for preliminary evaluation and refinement. Finally, the scale is administered in confirmatory testing, where a full psychometric evaluation is provided.*

Ethics and Dissemination: *The project was approved by the Queen Mary University of London Research Ethics Committee [Reference: 2024-0231-341] as the lead site and subsequently endorsed by the University of Manchester Research Ethics Committee. The YLS scale and results of its psychometric evaluation will be published openaccess. The protocol provided here will allow researchers to evaluate the final scale generated against the plans set out. We also encourage the use and adaptation of the protocol to develop age-sensitive loneliness scales for other populations.*

****

Reviewer: 2 - see attached
Dr. Lukas Novak, Palacky University Olomouc Comments to the Author:
Please find my comments for the authors in the attached file.

We have copied the review and included our response at the end of the document for your convenience.

****

Reviewer: 3
Dr. Phoebe McKenna-Plumley, Queen's University Belfast Comments to the Author:

Thank you for the opportunity to review this manuscript. This is a timely and valuable scale development eiort which will surely be of interest to a range of researchers and practitioners. The scale development process appears appropriate and has many strengths (the use of co-production with young people throughout and the focus on intensity, frequency, and duration of loneliness, for example) and the statistical plan seems generally thorough. I have some points for clarification which are outlined below; I hope that these can be helpful for the authors.

Thank you for your helpful feedback on the protocol. We address your comments below.

COMMENTS:

This topic is timely and the rationale behind creating this scale is strong. A wellconstructed scale for young people would be a valuable addition to research and practice around loneliness and it is very encouraging to see meaningful engagement with young people in creating this scale.

Page 4: "Three young people are coinvestigators on the project and have co-authored the protocol; young people's feedback is also integrated into the protocol via a Young Person Advisory Group. Finally, additional groups of young people contribute to the project during the summer, oiering their perspectives at diierent stages of the project": It is surely valuable to have these insights from young people throughout project development and conduct. However, this section doesn't make it very clear how the roles of these three groups (co-investigators, advisory group, and additional groups) diier – this information is expanded in the Discussion (page 13-14), so perhaps some of this could be added/moved to this initial description of the diierent roles.

Thank you for your suggestion. We now provide more information about these roles in the methods (page 7):

Patient and Public Involvement

The YLS is co-produced with young people from design to dissemination, ensuring the YLS reflects young people's experiences, needs, and priorities. Young people are reimbursed for their time contributing to the project. Young people are involved at the following levels of the project: IA and JC were co-investigators on the grant proposal, and IA, JC, and WW are

co-authors of this paper. They have contributed to all project stages by providing feedback and leading the co-production work. Around six young people are advisors in our young person advisory group, who give feedback on research design, materials, and dissemination. Two of these young people are seconded to our steering committee on a rotating basis to provide guidance and oversight. Additional groups of young people support ongoing work on the YLS as summer students, contributing, e.g., lived experience advice or supporting recruitment and the production of dissemination outputs.

Could the authors comment on their rationale for specifically focusing on 10–24-yearolds, rather than a narrower or wider age range? Relatedly, it seems that some workshop participants were younger (8-11 years) - is there a reason why some younger participants were also included in the development work but won't be considered a relevant age group for the final scale?

We thank the reviewer for this important question regarding our rationale for the target age range and the inclusion of younger participants in the development phase.

The final version of the Youth Loneliness Scale is intended for use with 10-24-year-olds, in line with widely accepted definitions of "youth" used by organizations such as the United Nations and the World Health Organization. This range captures the key developmental transitions between late childhood and adulthood - periods during which the experience, expression, and implications of loneliness may be both pronounced and developmentally distinct [1,2].

The decision to additionally include participants aged 8 and 9 in the item development phase was made on developmental and psychometric grounds.

We intentionally included some 8- and 9-year-olds in the early qualitative workshops to ensure developmental sensitivity and inclusivity in the early stages of scale design. Including younger voices helped us identify language and experiences that were comprehensible and relevant across a broader spectrum of ages. This strengthened the scale's capacity to resonate with the lower end of our target age group (10 - 12 years) – a key priority for us.

While data from 8- and 9-year-olds informed the content and phrasing of initial items, participants aged below 10 years were not included in the subsequent steps of the scale development process. Cognitive testing, exploratory, and confirmatory analyses have been and will be conducted with data from 10- to 24-year-olds only. Based on existing literature [3] and prior measurement studies [4], we recognise that children (under 10s) typically require diierent approaches to assessing loneliness, including diierent response formats, and context-specific items that align with their cognitive and emotional development.

We hope this clarifies the rationale for both the selected age range and the inclusion of younger participants in the early development phase. We have now included additional details in the manuscript, as follows (page 8):

Although the final scale is intended for use in 10- to 24-year-olds, we deliberately included some younger participants in this initial qualitative work. Including 8- and 9year-olds in the item-generation process helped us identify language and experiences that were comprehensible and relevant to young people across a broad age range. This strengthened

the scale's capacity to resonate with the lower end of our target age range (10 - 12 years), which can be particularly challenging [14,27].

Page 6: "The research team continually refined these items through group discussions at moderation meetings": It could be helpful to understand the types of decisions that were made through these discussions – i.e. what were the criteria leading to items being discarded at this stage?

We now describe the criteria we used to assess the items in more detail:

Page 8:

The research team continually refined these items through group discussions at moderation meetings [29]. Criteria used to assess items during these meetings were guided by DeVellis & Thorpe [21] and included combining similarly phrased items into a single item that captured the overarching meaning, ensuring items were straightforward to understand and appropriate for the target demographic, and applicable across all age groups. We also omitted items that were double-barrelled, required reverse-scoring, used double negatives, were likely to be 'leading', or were likely to elicit little variation in responses.

Perhaps the authors could briefly comment on characteristics of the young people who contributed to scale development beyond age and gender? This does not need to be exhaustive and confidentially is vital, but a narrative description of other demographic characteristics that were included/missing in these groups would be useful. For example, on page 8, it is noted that one item was removed as it was not applicable for neurodivergent youth – with this in mind, it would be useful to understand how diverse the overall group were in ways beyond age and gender, given that their insights were very instrumental to the development process.

We have now uploaded additional demographic information (including information about ethnicity and socioeconomic status) for participants in Steps 1- 3 here: https://osf.io/4qvnh/. To prevent statistical disclosure in reporting young people's demographics, we have collapsed data across stages and used the ONS 10-5 rule (suppressing counts < 10 and rounding to the nearest 5). For adults (N = 24), we provide a narrative summary.

Description in the main text (page 7):

The samples for Steps 1-3 were diverse in terms of ethnicity and somewhat skewed towards higher parental education and income levels but with reasonable absolute participant numbers across education and income brackets. There were more females than males overall. Detailed participant characteristics are reported in the Supplementary Information: https://osf.io/4qvnh/.

Supplementary Information**:**

Participant characteristics Step 1-3: *Young people participated in Steps 1-3. Adults participated in the Delphi survey only. Detailed participant characteristics for young people participants can be found in Supplementary Table 1. For adult participants, we provide a narrative summary here to prevent statistical disclosure with low sample sizes in sub-groups:*

Twenty-four professional experts (academics working on loneliness and/or youth development and/or psychometrics; and other stakeholders such as charities or practitioners working with young people) participated in the Delphi survey. In the professional expert group, the number of years they had worked in their profession ranged from ~0-40. Around half were currently working in the UK, and the remaining participants were working in a range of countries across the globe. The age range of this group was ~25-70 years, approximately 60% identified as female, and approximately 90% identified as White.

Supplementary Table 1. Young people participant demographics.

Demographic Variable	***~N***
Location	
Manchester area[a]	*55*
London area[a]	*75*
Online	*95*
Age group	
10-11[b]	*35*
12-15	*65*
16-18	*70*
19-24	*45*
Missing	***
Sex	
Male	*70*
Female	*140*
Prefer not to say	***
Missing	*10*
Gender	
Male	*70*
Female	*135*
Non-binary	***
Prefer not to say	***
Missing	***
Ethnicity	
White	*100*
Asian or Asian British	*50*
Black, Black British, Caribbean or African	*30*
Mixed or multiple ethnic groups	*15*
Other ethnic group	***
Prefer not to say	***
Missing	***
Parent/Guardian highest qualification	
No qualifications	*20*
Below degree level	*60*
Degree or equivalent	*65*

Above degree level	*45*
Not known	***
Prefer not to say	***
Missing	*10*
Parent/Guardian total annual income	
Below £10,000	*20*
£10,001-£20,000	*30*
£20,001-£30,000	*30*
£30,001-£40,000	*15*
£40,001-£50,000	*15*
Above £50,001	*50*
Not known	*10*
Prefer not to say	*30*
Missing	*25*

Note: Counts are collapsed across Steps 1-3 to prevent statistical disclosure. Additionally, cells with counts < 10 are suppressed (*), and the remaining cells are rounded to the nearest 5 to prevent statistical disclosure. [a]Data refers to in-person workshops and focus groups only. [b]Age 8-11 for the in-person workshops and focus groups.

Page 9: "Both groups recommended asking about the time frame of the 'past month'. In terms of the most important dimension to ask about, both groups recommended asking about intensity as the most important, followed by frequency.": For readers unfamiliar with other loneliness measures, it might be helpful to comment briefly on how much this aligns or diiers from other common loneliness scales and whether the authors anticipate any implications for making comparisons with other youth scales or adult loneliness measures.

We appreciate the reviewer's observation. As noted by the reviewer, the inclusion of duration and a focus on intensity reflects direct input from young people and stakeholders in our development process.

This approach represents a notable departure from most existing loneliness measures, particularly those used with youth. Commonly used tools, such as the UCLA Loneliness Scale and its derivatives, primarily assess the frequency or persistence of loneliness-related feelings or behaviour (e.g., "How often do you feel left out?"), typically using Likert-type scales (e.g., "never" to "often"). These measures rarely include reference to timeframe, limiting contextual precision [5].

Moreover, intensity - the subjective severity or emotional impact of loneliness - is almost entirely absent from existing youth and adult loneliness scales [4,5]. Yet intensity oiers important additional insight: it helps distinguish between transient or low-impact experiences and those that are more distressing and potentially clinically relevant. For instance, a young person may experience loneliness frequently but not find it troubling, whereas another may have infrequent but highly distressing experiences.

We now make this issue explicit in the Introduction (page 5):

(…), loneliness is usually probed only through ratings of frequency (e.g., "How often do you feel lonely?") with little reference to timeframes, limiting contextual precision [5], and limited consideration of other important dimensions [10], such as duration, intensity, and emotional impact.

By incorporating both duration and intensity, our measure aims to enhance clinical and conceptual sensitivity, particularly in identifying individuals for whom loneliness constitutes a meaningful problem.

We recognise that this may introduce some challenges for direct comparison with existing youth or adult measures, which typically focus on frequency without duration or intensity. However, we believe the benefits outweigh those limitations, and our approach provides a foundation for future harmonisation work.

Could the authors comment on their decision to have items focusing on specific relationship types (family/caregiver and friends) within the items, rather than having all non-specific items (like "I don't feel wanted")? Some of these items may be hard to respond to as they depend on the availability of these relationships (e.g. "I feel ignored by friends" - a 'Strongly disagree' response could mean that a person is satisfied with their friends or that they don't have friends to feel ignored by). I appreciate that these relationships were considered most important by the expert groups, but perhaps it is worth commenting on the strengths and weaknesses of including specific relationships within the scale.

Thank you, this is an important point, and one we discussed at length as a team. We decided to include diierent relationship types for several reasons. First, feedback received from our expert groups suggested that items that were non-specific and referred to 'others' or 'people in my life' were sometimes reported to be too abstract, and harder to understand and respond to than ones that specified the nature of the relationship. Second, covering diierent relationships is useful for future developmental research which suggests changes in these relations between childhood and adulthood [6,7]. Third, including information on loneliness in diierent types of relationships provides opportunities for informing prevention and intervention work. To balance the drawbacks of including specific dimensions, as noted by the reviewer, we made items as inclusive as possible, e.g. using the labels 'family/caregivers'. Finally, we ensured that we also included a set of non-specific items, resulting in a mixture of relationship-specific and non-specific items.

We now acknowledge this issue in the strengths and limitations section (page 2):

- *The YLS covers both relationship-specific items (friends, family/caregivers) and general items. This balances the need for concrete items that can inform research and practice, with the need for general items that may be particularly useful for short scales.*

We also elaborate on our approach in the Methods and Analysis section (page 11):

In quantitative and qualitative feedback, experts by experience reported friends and family as the most important relationships to ask about. They also noted that these items are concrete and easy to understand. Professional experts preferred the questionnaire to include

general items that do not reference specific relationships. To balance these demands, we included both concrete items referring to friends and family/caregivers and general items.

The planned EFA and CFA processes appear to be generally thorough and appropriate. Some additional information would be valuable on certain points - perhaps the authors could specify the values that would constitute high cross-loading or poor loading items, and the factor extraction method and rotation for EFA.

Thank you for this suggestion. We have now specified the factor extraction and rotation techniques, as well as thresholds for considering item removal.

Page 13:

For this, we will use parallel analysis, as implemented in the psych package [36] in R: fa.parallel(data, fa = "fa", n.iter = 100). We will repeat this analysis with polychoric correlations as a robustness check [37]. The results of fa. parallel will also be compared to the CD and SMT methods implemented in EFAtools [38,39] as a robustness check. The EFA will then be fit with rotate = "oblimin", implementing an oblique rotation, to allow for correlations between factors [40]. Cross-loading items or items with poor loadings [41] will be removed unless there is a strong theoretical rationale for keeping them.

The following criteria will be applied to determine items for consideration for removal:

- *Items with Cronbach's alpha below 0.7 and inter-item correlations below 0.2 [42].*
- *Items with Cronbach's alpha above 0.9 (which may indicate redundancy) [43].*
- *Items with an item-discrimination index below 0.2 [42].*
- *Items with an item-total correlation below 0.3 [44]*
- *Items with factor loadings below 0.4 [40]*
- *Items with cross-loadings above 0.3 [40]*
- *Items with a diyerence between the loading on the primary and alternative factors below 0.2 [40].*

In Table 3, could the authors include a specification of the direction and potentially the magnitude of the correlations they expect to observe between these variables and the YLS to decide that convergent and divergent validity have been achieved?

Thank you for this suggestion. We now include a specification of our predicted direction and magnitude.

Page 16:

A strong correlation between scales (r > 0.5) will be taken to indicate convergent validity of scales measuring the same construct [52]. A correlation below 0.9 between measures of diyerent constructs will be taken to suggest acceptable discriminant validity [53]. We expect positive strong correlations between the YLS and other measures of loneliness. We expect positive, but weaker correlations between the YLS and measures of depression, general anxiety, social anxiety and introversion. We expect negative and weaker correlations between the YLS and well-being.

The Discussion is thorough and clearly identifies the strengths and need for the authors' approach, as well as guiding future work in terms of scale adaptation.

I hope these comments can be useful for the authors.

****
****

Reviewer: 2 – copied here for convenience Dear Editor, thank you very kindly for the opportunity to review this protocol. I think that the development of a new loneliness measure focused on assessing loneliness in young people can be a meaningful contribution to the field. Overall, the quality of the protocol is very high. However, there are several points I invite authors to think about or incorporate into their protocol to potentially improve the quality of the resulting study. Thank you for your constructive feedback on our paper. We respond point-by-point below.

First, the authors state that: the „Youth Loneliness Scale (YLS) project's overarching objective is to develop, evaluate, and disseminate a developmentally sensitive measure of loneliness for young people aged 10-24 years living in the UK. We select this broad age range to allow us to track age-linked continuities and changes in how loneliness is experienced across the full set of developmental transitions in youth." Although I understand the author's intention to capture the degree of loneliness across a wide age group of young people, I think that it would be desirable to provide more arguments for the lower and upper bound of the selected age group of interest. In more detail, why 10 and not e.g. 8 years old children, are creating a lower bound of the age group of interest, and why do 24 and not 26 form the upper bound of the age group of interest?

Thank you for this question. The final version of the Youth Loneliness Scale is intended for use with 10-24-year-olds, in line with widely accepted definitions of "youth" used by organizations such as the United Nations and the World Health Organization. This range captures the key developmental transitions between late childhood and adulthood - periods during which the experience, expression, and implications of loneliness may be both pronounced and developmentally distinct [1,2].

The decision to additionally include participants aged 8 and 9 in the item development phase was made on developmental and psychometric grounds.

We intentionally included some 8- and 9-year-olds in the early qualitative workshops to ensure developmental sensitivity and inclusivity in the early stages of scale design. Including younger voices helped us identify language and experiences that were comprehensible and relevant across a broader spectrum of ages. This strengthened the scale's capacity to resonate with the lower end of our target age group (10 - 12 years) – a key priority for us.

While data from 8- and 9-year-olds informed the content and phrasing of initial items, participants aged below 10 years were not included in the subsequent steps of the scale development process. Cognitive testing, exploratory, and confirmatory analyses have been and will be conducted with data from 10- to 24-year-olds only. Based on existing literature [3] and prior measurement studies [4], we recognise that children (under 10s) typically

require diierent approaches to assessing loneliness, including diierent response formats, and context-specific items that align with their cognitive and emotional development.

We hope this clarifies the rationale for both the selected age range and the inclusion of younger participants in the early development phase. We have now included additional details in the manuscript, as follows (page 8):

Although the final scale is intended for use in 10- to 24-year-olds, we deliberately included some younger participants in this initial qualitative work. Including 8- and 9year-olds in the item-generation process helped us identify language and experiences that were comprehensible and relevant to young people across a broad age range. This strengthened the scale's capacity to resonate with the lower end of our target age range (10 - 12 years), which can be particularly challenging [14,27].

Second, in Step 4, the authors say: „The survey will be administered twice, two weeks apart, to assess test-retest reliability." What rationale do authors have for selecting a two-week period for test-retest and not, e.g., one week?

That is a good question. Our read of the literature is that there is currently little definitive guidance on designing test-retest reliability assessments. The advice is generally to space assessments suiiciently far apart to avoid memory eiects but not too far, to avoid sampling systematic change in the construct, with optimal spacing dependent on the construct of interest [8–10]. As memory eiects may be more likely to occur within around 0-14 days post the first measurement [10,11], we opted for a 2week interval.

We have now added relevant references to the text (page 12):

The survey is administered twice, two weeks apart [32,33], to assess test-retest reliability.

Third, the authors say that: „Problematic items with low reliability, variability or consistency with other items will be removed." I understand that it might be challenging to set up thresholds for some metrics, e.g., variability of scores and that to decrease researchers' degrees of freedom, it might be advisable to define thresholds for, e.g., internal consistency, factor loadings, etc. This might increase transparency for item selection in step 4. I would also ask authors to specify more clearly, what the authors mean by poorly performing dimensions.

Thank you, we agree with this suggestion. We have now specified thresholds for identified items for consideration for removal. We have also clarified what we mean by poorly performing dimensions: Where the resulting scale yields a solution with fewer than four items per factor, we will consider removing that factor.

Page 13:

The following criteria will be applied to determine items for consideration for removal:

- *Items with Cronbach's alpha below 0.7 and inter-item correlations below 0.2 [42].*
- *Items with Cronbach's alpha above 0.9 (which may indicate redundancy) [43].*
- *Items with an item-discrimination index below 0.2 [42].*

- *Items with an item-total correlation below 0.3 [44]*
- *Items with factor loadings below 0.4 [40]*
- *Items with cross-loadings above 0.3 [40]*
- *Items with a diyerence between the loading on the primary and alternative factors below 0.2 [40].*

Where the resulting scale yields a solution with fewer than four items per factor, we will consider removing that factor. This is because a factor with fewer than four items can pose issues with identification and reliability [44].

Fourth, the authors state that: „Exploratory Factor Analysis (EFA) will be employed to test the dimensionality of the scale and further identify poorly performing items and/or dimensions for removal. For this, we will use parallel analysis, as implemented in the psych package [32] in R.“ Although I am very happy that the authors specified what method they will use to extract a number of factors, I think that some additional arguments for using parallel analysis must be provided – especially given the fact that there exist many methods for factor extraction (see the following simulation study for more details: https://doi.org/10.1037/met0000200). Relatedly, I would recommend authors use at least two methods for factor extraction because it adds credibility to a number of extracted factors if results from both methods will yield the same results. On the OSF page, I could not find code for the simulation study determining minimal sample size. Please consider sharing your simulation code.

Thank you for drawing our attention to this issue. We have now specified that we will compare results from fa.parallel with alternative correlation and extraction methods as a robustness check.

Page 13:

Exploratory Factor Analysis (EFA) will be employed to test the dimensionality of the scale and further identify poorly performing items and/or dimensions for removal. For this, we will use parallel analysis, as implemented in the psych package [36] in R: fa.parallel(data, fa = "fa", n.iter = 100). We will repeat this analysis with polychoric correlations as a robustness check [37]. The results of fa. parallel will also be compared to the CD and SMT methods implemented in EFAtools [38,39] as a robustness check. The EFA will then be fit with rotate = "oblimin", implementing an oblique rotation, to allow for correlations between factors [40].

We have also uploaded our scripts for sample size calculations to the OSF: https://osf.io/4qvnh/

Fifth, the authors say: „Test-retest reliability will be assessed using the Intraclass Correlation Coeiicient (ICC) using irr. “ I appreciate very much that the authors are specific regarding how ICC will be calculated. I would however, strongly recommend to authors what type of ICC will be used since this can be definitely specified in advance. See this canonical article for more details: https://doi.org/10.1016/j.jcm.2016.02.012

We have now specified the type of ICC we will use (page 14):

Test-retest reliability will be assessed using the Intraclass Correlation Coeyicient (ICC) using irr [46] with the following specifications: icc(ratings, model = c("twoway"), type = c("agreement"), unit = c("single")) [47].

Sixth, authors states: „invariance will be assessed to determine the factor structure indiierent age groups and genders." I would recommend including the exact age categories that will be tested in MI. Relatedly, what reasons do authors have for omitting scalar invariance testing?

We now specify the age groups for measurement invariance testing and clarify that we plan to assess configural, metric and scalar measurement invariance.

Page 14:

Configural, metric and scalar measurement invariance will be assessed to determine the factor structure in diyerent age groups (10-11 years, 12-15 years, 16-18 years, 1924 years) and genders. Nested models will be compared using the delta chi-square, RMSEA and CFI using the criteria set out in [51].

Finally, the authors expect that the scale will have three dimensions. This expectation needs to be based on some theoretical framework underlying this expectation. Thus, I suggest that the authors state what theoretical framework guided them to think about their scale as having three dimensions.

Thank you for this comment. We think the reviewer is referring to our decision to use the three dimensions of duration, frequency and intensity as the response options upon which each item is assessed. We used these three dimensions as they are most frequently used to diierentiate diierent loneliness experiences, especially ones that are associated with more severe forms of loneliness. In a recent systematic review of how researchers conceptualise "chronic" loneliness, these dimensions were used most often to describe variability in the loneliness experience [12]. In our empirical work, we have also used these three dimensions to assess individual diierences in loneliness experiences [13]. If we have misinterpreted what the reviewer means by dimensions, please let us know and we can clarify this further.

We have now added relevant references to justify investigating the frequency, duration and intensity of loneliness (page 9):

Loneliness was also thought to vary in intensity, duration, and frequency; in line with previous accounts of loneliness [20,31].

Relatedly, please explain (at least to me in your response) why you think that a common cause theory will generate your data and why you do not think about relationships between items as they are mutually interacting with each other in a network. See e.g., this article: https://doi.org/10.1080/1047840X.2020.1853461.

Thank you for this question. We agree that network models of psychopathology and related constructs are often compelling. We have used them in previous work [14]. We think the use of latent variable approaches here is defensible for several reasons, however.

First, a reflective model is not implausible – items of the YLS can be thought of as diierent expressions of the same underlying construct (loneliness).

Second, latent variable approaches allow comparing the psychometric properties of the YLS with those of other scales (typically evaluated via latent variable models). This will be important to potential users of the YLS.

Third, network models are mathematically equivalent to latent variable models [15] and using latent variable models for psychometric evaluation does not preclude future use of the YLS in network models.

Future empirical work will be able to shed more light on the underlying processes that give rise to the experience of loneliness.

****

References

1 Goossens L, Victor C, Qualter P. Introduction to the special section on loneliness across the life span. *International Journal of Behavioral Development*. 2022;46:1–2. doi: 10.1177/01650254211029719

2 Matthews T, Qualter P, Bryan BT, *et al.* The developmental course of loneliness in adolescence: Implications for mental health, educational attainment, and psychosocial functioning. *Dev Psychopathol*. 2023;35:537–46. doi: 10.1017/S0954579421001632

3 Robyn Cole A, Bond ,Caroline, and Qualter P. Primary-school aged children's understanding and experiences of loneliness: a qualitative enquiry. *Pastoral Care in Education*. 2023;41:63–83. doi: 10.1080/02643944.2021.1977993

4 Cole A, Bond C, Qualter P, *et al.* A Systematic Review of the Development and Psychometric Properties of Loneliness Measures for Children and Adolescents. *International Journal of Environmental Research and Public Health*. 2021;18. doi: 10.3390/ijerph18063285

5 Qualter P, Petersen K, Barreto M, *et al.* Exploring the Frequency, Intensity, and Duration of Loneliness: A Latent Class Analysis of Data from the BBC Loneliness Experiment. *International Journal of Environmental Research and Public Health*. 2021;18. doi: 10.3390/ijerph182212027

6 Blakemore SJ, Mills KL. Is adolescence a sensitive period for sociocultural processing? *Annual Review of Psychology*. 2014;65:187–207. doi: 10.1146/annurevpsych-010213-115202

7 Nelson EE, Leibenluft E, McCLURE EB, *et al.* The social re-orientation of adolescence: a neuroscience perspective on the process and its relation to psychopathology. *Psychological Medicine*. 2005;35:163–74. doi: 10.1017/S0033291704003915

8 Marx RG. Comparing time intervals for test-retest reliability: measuring stability and calculating a sample size. *Journal of Clinical Epidemiology*. 2004;57:995. doi: 10.1016/j.jclinepi.2004.03.007

9 Aldridge VK, Dovey TM, Wade A. Assessing test-retest reliability of psychological measures: Persistent methodological problems. *European Psychologist*. 2017;22:207–18. doi: 10.1027/1016-9040/a000298

10 Park MS, Kang KJ, Jang SJ, *et al.* Evaluating test-retest reliability in patient-reported outcome measures for older people: A systematic review. *International Journal of Nursing Studies*. 2018;79:58–69. doi: 10.1016/j.ijnurstu.2017.11.003

11 Clinical: Retesting Time Advice for Clinical Assessments. https://support.pearson.com/usclinical/s/article/Clinical-Customer-Support-TestRetest-Minimum-Time-Advice (accessed 4 June 2025)

12 Conneely M, Zheng Y, Medlicott E, *et al.* Defining and understanding the next global mental health challenge: chronic loneliness. 2025.

13 Qualter P, Petersen K, Barreto M, *et al.* Exploring the Frequency, Intensity, and Duration of Loneliness: A Latent Class Analysis of Data from the BBC Loneliness Experiment. *Int J Environ Res Public Health*. 2021;18:12027. doi: 10.3390/ijerph182212027

14 Pollmann A, Fritz J, Barker E, *et al.* Networks of Adversity in Childhood and Adolescence and Their Relationship to Adult Mental Health. *Research on Child and Adolescent Psychopathology*. Published Online First: 4 November 2022. doi: 10.1007/s10802-022-00976-4

15 van Bork R, Rhemtulla ,Mijke, Waldorp ,Lourens J., *et al.* Latent Variable Models and Networks: Statistical Equivalence and Testability. *Multivariate Behavioral Research*. 2021;56:175–98. doi: 10.1080/00273171.2019.1672515

VERSION 2 - REVIEW

**Reviewer**	**2**
**Name**	**Novak, Lukas**
**Affiliation**	**Palacky University Olomouc, Social Health Institute**
**Date**	**24-Jun-2025**
**COI**	

I am satisfied with the implementation of my comments by the authors.
